# Persistent Valence Representations by Ensembles of Anterior Cingulate Cortex Neurons

**DOI:** 10.3389/fnsys.2018.00051

**Published:** 2018-10-17

**Authors:** Barak F. Caracheo, Jamie J. S. Grewal, Jeremy K. Seamans

**Affiliations:** Djavad Mowafaghian Centre for Brain Health and Department of Psychiatry, Faculty of Medicine, University of British Columbia, Vancouver, BC, Canada

**Keywords:** neurons, electrophysiology, ensembles, prefrontal cortex, rats, tetrodes, emotion, pavlovian

## Abstract

The anterior cingulate cortex (ACC) responds to outcomes of a positive or negative valence, but past studies typically focus on one valence or the other, making it difficult to know how opposing valences are disambiguated. We recorded from ACC neurons as rats received tones followed by aversive, appetitive or null outcomes. The responses to the different tones/outcomes were highly inter-mixed at the single neuron level but combined to produce robust valence-specific representations at the ensemble level. The valence-specific patterns far outlasted the tones and outcomes, persisting throughout the long inter-trial intervals (ITIs) and even throughout trial blocks. When the trials were interleaved, the valence-specific patterns abruptly shifted at the start of each new trial. Overall the aversive trials had the greatest impact on the neurons. Thus within the ACC, valence-specificity is largely an emergent property of ensembles and valence-specific representations can appear quickly and persist long after the initiating event.

## Introduction

Most laboratory tasks are motivated by either pleasure or pain and as such are endowed with motivational valence. Human imaging studies have shown that the medial frontal cortex (MFC) including the anterior cingulate cortex (ACC) is activated during the experience of pain, fear and sadness (Peterson, 1986; Kulkarni et al., 2005; Wiech and Tracey, 2009; Etkin et al., 2011; Shackman et al., 2011; Yoshino et al., 2013; Palermo et al., 2015; Wagner et al., 2015; Fullana et al., 2016) as well as by the experience of pleasure or happiness (Rolls et al., 2003; Lindgren et al., 2012; Matsunaga et al., 2016; Suardi et al., 2016). At the cellular level, MFC/ACC neurons respond robustly to cues that predict aversive outcomes. This includes Pavlovian tone-electrical eyelid stimulation conditioning in rabbits (Powell et al., 1990, 1996; Powell and Ginsberg, 2005) and Pavlovian tone-footshock conditioning rats (Baeg et al., 2001; Burgos-Robles et al., 2009; Halladay and Blair, 2015). MFC/ACC neurons also respond robustly to cues that predict reward or appetitive outcomes in primates (Shidara and Richmond, 2002; Amiez et al., 2006; Kennerley and Wallis, 2009; Hayden et al., 2011; Cai and Padoa-Schioppa, 2012; Toda et al., 2012; Khamassi et al., 2015) and in rats (Pratt and Mizumori, 2001; Petykó et al., 2009, 2015).

Currently it remains an open question how opposing valences are disambiguated by the ACC since past studies have typically focused on the responses of ACC neurons to outcomes of one valence or the other. One possibility is that opposing valences are processed by unique subregions of the ACC or by valence-specific neurons. Alternatively, because MFC neurons exhibit a high degree of mixed-selectively (Woolgar et al., 2011; Heilbronner and Hayden, 2016; Ma et al., 2016), another possibility is that all neurons are more or less responsive to both valences and valence-specificity only emerges at the ensemble level.

The goals of the present study were to investigate how opposing valences are represented by ACC neurons and to explore the temporal characteristics of valence representations. A simple task design was employed whereby food deprived rats, implanted with tetrode arrays aimed at the ACC were presented with three unique auditory tones followed by either an appetitive (food pellet), aversive (footshock), or neutral (nothing) outcome. The trials were separated by long (~50 s) inter-trial intervals (ITIs) and were presented in either a blocked or inter-leaved format. Consistent with the results of past studies, robust responses to the tones and outcomes were observed. However, strictly valence-specific neurons were rare as most neurons were responsive to some mixture of the three tones and outcomes. Highly distinct, valence-specific patterns did emerge at the ensemble level and were found to persist throughout the long ITIs. When the trials were presented in an interleaved manner, the ensembles shifted back and forth between the three valence-specific patterns and the shifts were largest if the current outcome was aversive. These findings provide new insights into how the ACC encodes valence-specific information.

## Materials and Methods

### Experimental Subjects

Eight male Long-Evans rats (Charles River Laboratories, Montreal, QC, Canada) weighing between 400 g and 470 g were used. They were housed in an inverted 12-h day/12-h night cycle and were food restricted to 90% of their free-feeding weight but given unlimited access to water for the duration of training. Tetrode array implant surgery was performed before training began. All procedures were conducted in accordance with the Canadian Council of Animal Care and approved by the Animal Care Committee of the University of British Columbia (A14-0084).

### Behavior

Recording and training sessions took place inside a custom made behavioral chamber (30 cm × 25 cm × 60 cm) with a pellet dispenser (dispensing 45 mg Bioserv sweet, dustless precision pellets into a magazine cup), a shock grid and a tone emitting speaker that were all connected to a Med Associates (St. Albans, VT) control box and to a PC workstation running Med-PC software (St. Albans, VT). Before training began, rats were habituated to the chamber and accustomed to eating food from the magazine cup. They were also exposed to foot shocks in order to determine the intensity of the shocks for the experiments. The shock duration was 500 ms and the intensity was varied between 0.5 mA and 0.7 mA and was chosen based on the minimum level required to elicit a noticeable behavioral response. After rats had initial exposure to the chamber and to the two outcomes, tone conditioning began. Three distinct frequency (4, 6, 8 kHz) tones were assigned to three possible outcomes: food (F), shock (S) or nothing (N). Prior to conditioning, the tones evoked no noticeable behavioral reactions. Tones were randomly assigned to an outcome for each rat, but once conditioning began, tone-outcome contingencies remained unchanged for the duration of the experiments. During the initial training period, tone-food pairings were conducted for 5 days, followed by 2–3 days of tone-shock and tone—no outcome pairings. Rats were exposed to at least three sessions where they received all three contingencies in consecutive blocks before any of the recordings used in this study took place. A session consisted of three blocks with the order randomly selected from all possible combinations. Each block consisted of 30 trials where the tone was presented for 5 s and the outcome was delivered immediately thereafter. The average ITI was 49 s (range 25–85 s). The data set for the block sessions was constructed from 15 recording sessions obtained from four animals and involved the following block orders: N-F-S (3); N-S-F (2); F-S-N (3); F-N-S (1); S-F-N (2); S-N-F (4). The four animals used in the interleaved sessions were trained in the same manner except that after the block sessions, they received multiple days where they were exposed to the three tones-outcome pairings in a randomized order (separated by an average ITI of 42.5 s, range 35–50 s).

**Figure 1:**
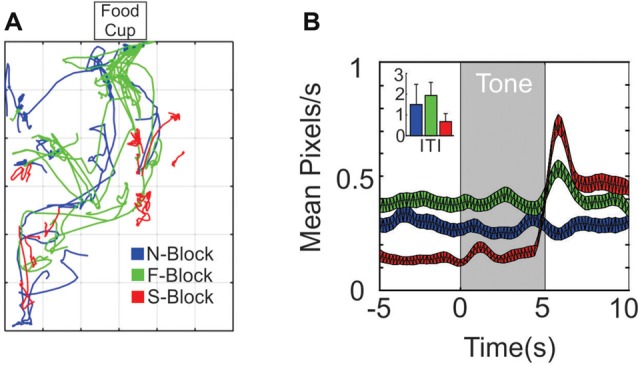
Differences in movement across the three blocks. **(A)** 10 × 10 s sample movement trajectory segments from the N-block (blue), the F-block (green) and the S-block (red) as obtained from video tracking during a single session. **(B)** Average (and SEM) rate of movement (pixels/sec) across all animals and sessions during the tone and outcome periods or (inset) during the inter-trial/background periods.

The animal’s behavior in each block was video recorded using an overhead camera. The x-y position of the head was tracked and was scaled to a square perimeter of 30 × 30 normalized pixels (Figure 1). In order to quantify movement, the pixels traversed in the 10 s period preceding the tone were compared to pixels traversed during the tone period using three time bin sliding window Kolmogorov-Smirnoff (KS) tests. In Figures 8C,D movement was quantified based on the distance vector between the xy position of the head in adjacent time bins.

**Figure 2:**
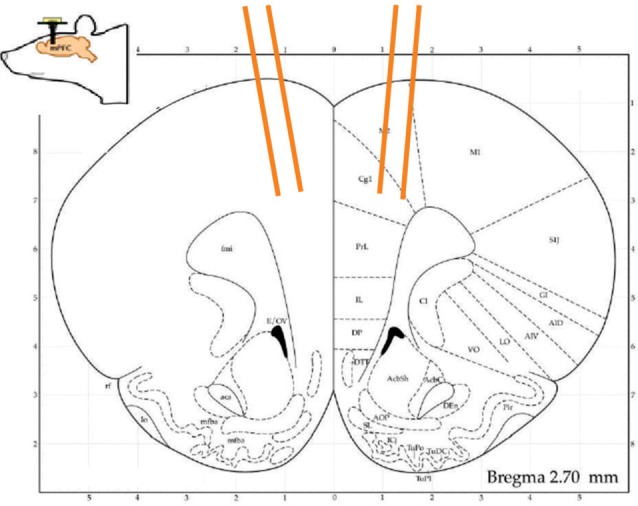
A schematic of the presumed tetrode tracts at 2.7 mm AP.

**Figure 3:**
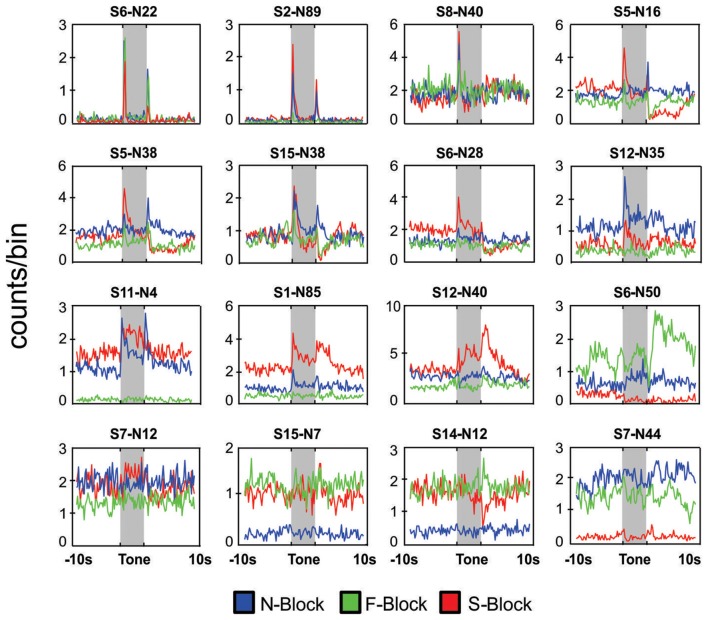
Neuronal responses in block sessions. Peri-event histograms of neuronal responses during a 20 s period centered on tone onset for 16 different neurons that were each recorded during an N-block (blue), F-block (green) and S-block (red). In each case the tone lasted for 5 s (shaded area) and was immediately followed by the outcome. Placement of the panels was based on the type of response: neurons that responded mainly the tones or the outcomes are presented in the top rows whereas those exhibiting strong block offsets are presented in the bottom rows. The numbers above each panel denote the session and neuron number.

**Figure 4:**
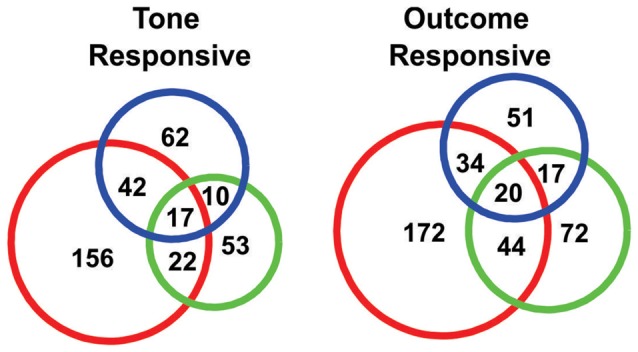
Venn diagrams showing the numbers of “tone responsive” (left) and “outcome response” (right) neurons.

**Figure 5:**
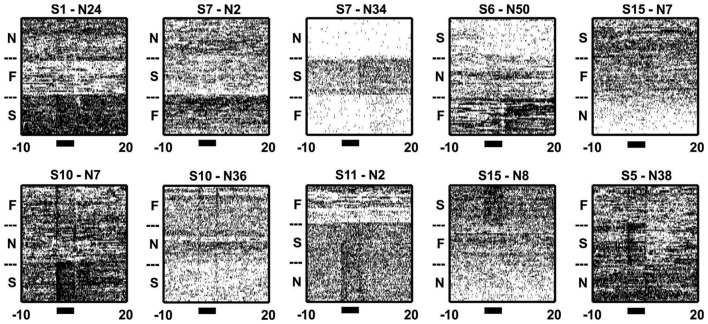
Inter-trial/background activity differed across blocks. Sample raster plots from 10 neurons illustrating the changes in activity during trial and inter-trial periods of different blocks. Note how the neurons changed their firing throughout both the trial (black bars) and/or inter-trial periods of the blocks. The subheadings give the session and neuron numbers, while the N, F and S denotes N-block, F-block and S-block, respectively.

**Figure 6:**
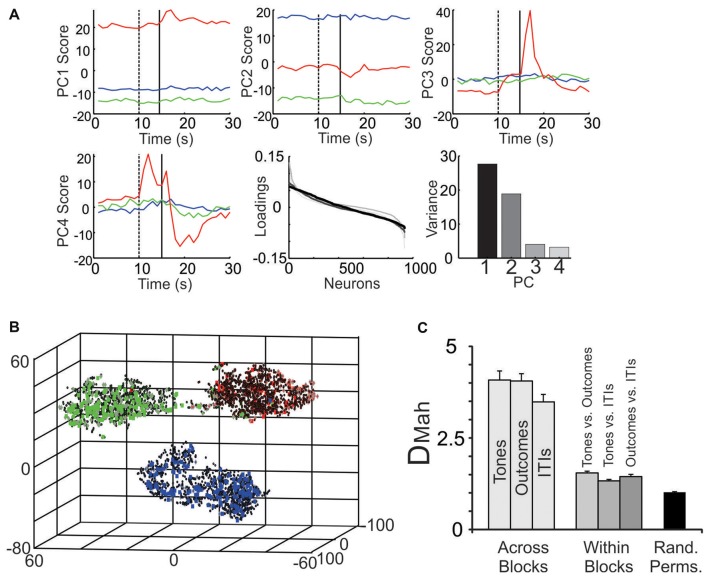
Parsing block-specific ensemble dynamics. **(A)** Decomposition of ensemble signals using PCA. When PCA was performed on all 934 neurons, PC1 (top left) and PC2 (top middle) showed strong block offsets, while PC3 (top right) and PC4 (bottom left) exhibited fluctuations mainly during the tone and outcome periods, especially of the S-block. In all panels PCs during the S-block are in red, the F-block in green and the N-block in blue. The dotted vertical line denotes tone onset and the solid line denotes outcome onset. (Bottom middle) The loadings of the neurons on the top four PCs. The loadings are color-coded based on their ranking starting with PC1 (black) and going to PC4 (light gray). Note that in this figure, the relative positions of the neurons on the x-axis varied by PC. (Bottom right) The portion of variance accounted for by each PC. **(B)** The multiple single unit activity (MSUA) space from a single session was reduced to three dimensions for visualization using t-distributed stochastic neighbor embedding (t-SNE). Each point in the space represents the activity of the ensemble during a single 1 s time bin. The gray diamonds are derived from time bins when tones were presented, the squares from time bins when the outcomes were delivered (specifically, the 0–5 s following the tones) and the black dots from time bins associated with the inter-trial intervals (ITIs). Each symbol is filled based on the block from which it was derived (F-block = green, the N-block = blue, S-block = red). **(C)** The average Mahalanobis distance (D_Mah_ and SEM) between the clusters of points corresponding to time bins associated with tone, outcome or ITI periods derived from different blocks (light gray bars), from within a single block (dark gray bars) or from time bins taken at random from throughout the task (black bar; Rand. Perms. = random permutations). Each light gray bar involves an averaging over all combinations of block pairs (N vs. F; N vs. S; F vs. S blocks) while all darker gray bars average over all blocks.

**Figure 7:**
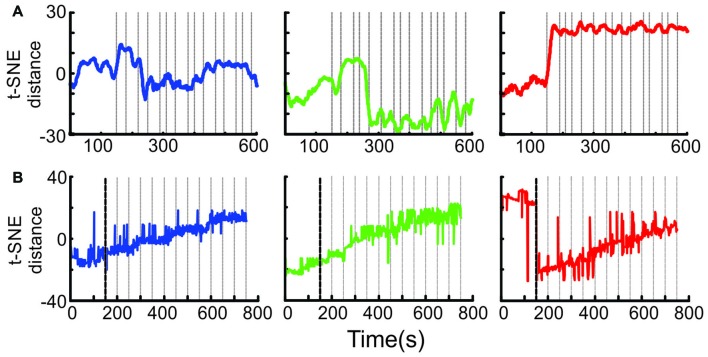
Ensemble transition dynamics. **(A)** The full MSUA space from a single session (*n* = 65 neurons) was reduced to a single dimension using t-SNE which provided a distance metric of how the ensemble activity changed through time starting from the 150 s period preceding block onset through the first 15 trials of the N-block (left, blue), F-block (middle, green) or S-block (right red). The gray lines denote the time of tone onset. The raw t-SNE data was smoothed by a 20 bin rolling average. **(B)** The full multi-dimensional MSUA space that included all neurons in 13 sessions was reduced to a single dimension using t-SNE. Since multiple sessions with different trial times were combined, the gray lines represent the average time of tone onset (i.e., every 50 s).

**Figure 8:**
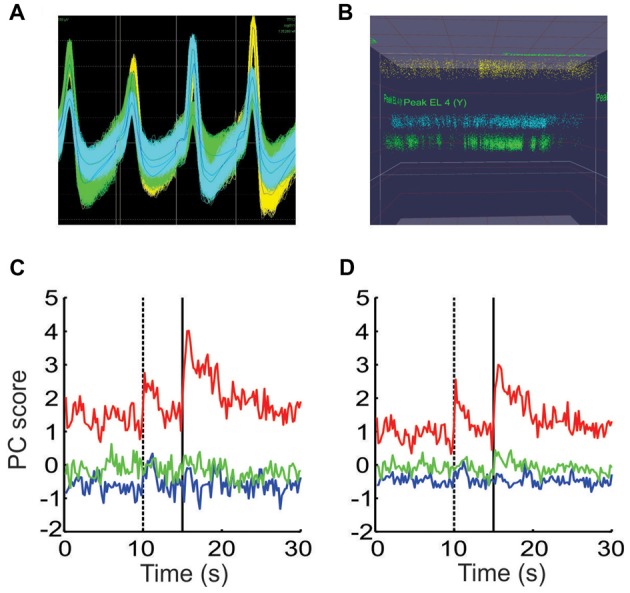
Controlling for other potential causes of the shifts in ensemble dynamics. **(A)** The waveforms of all spikes from three putative neurons recorded from a single tetrode. **(B)** A plot of the peak height of all spikes recorded on two wires throughout the session, using the same colors as in **(A)**. The peak height of the waveforms remained stable while the density of points fluctuated throughout the session. **(C)** PCA performed on the spike count data from a single session using the raw spike counts. **(D)** PCA performed on a matrix composed of the remaining portion of each neuron’s spike counts that were not accounted for by the animal’s movements.

### Surgery and Data Acquisition

Rats were surgically implanted with custom built 16 tetrode hyperdrive arrays (Figure 2). They were anesthetized under iso-flurane gas, their skull was surgically exposed and a 4 mm × 3 mm hole was drilled at +3.0 mm from Bregma and at least ±0.5 mm from the midline. The implant was positioned over the area and fixed to the skull with 11 skull screws and dental acrylic. Two additional screws used as ground wires were placed in the posterior skull. Tetrodes were lowered ~1,000 μm on the day of surgery and then the rats were given 1–2 weeks of recovery. Tetrodes were advanced up to 1,000 μm more to their target prior to the first recording session. In between sessions, tetrode drives were turned between 20 μm and 50 μm to maximize the units recorded and obtain different populations. When the experiments ended, rats were perfused and their brains collected and sliced on a cryostat. Slices were mounted on slides and viewed under a microscope to confirm the anatomical locations of tetrode tracts. Based on tetrode advancement records, the positions were estimated to have been mostly in the medial wall, within the ACC up to the border of the prelimbic cortex (PL).

Tetrodes were attached to EIB-36TT boards, plugged into two HS-36 headstages and connected via tether cables to a Digital Lynx 64-channel system (Neuralynx, Bozeman, MT) and then to a PC workstation. Electrophysiological data and behavioral events were captured using Cheetah 5.0. (Neuralynx, Bozeman, MT) Files were exported into Offline Sorter (Plexon, Dallas, TX) and were manually sorted based on three-dimensional projections of wave form peaks, valleys and principal components. All sessions were independently sorted by two individuals. Once consensus was achieved, spike waveform components on each wire were tracked through time to establish whether stability throughout the session was achieved. Only units whose waveform characteristics remained stable through the session were included in the study. Spike timestamps were then exported to Neuroexplorer 4, (Nex Technologies, Colorado Springs, CO) and to Matlab (Natick, MA) for further analysis.

### Data Analysis

#### Single-Unit Analyses

KS tests were performed in order to determine whether individual units were responsive to tones or outcomes. For these analyses, spike counts across the entire session were first normalized to values between 0 and 1 and tone (initial 1–2 s) and outcome (0–5 s from tone offset) responses were evaluated relative to a 10 s period preceding each tone. Responsiveness during the inter-trial or “background” period (i.e., all time bins excluding those from tone onset to 15 s after tone onset) was evaluated relative to the 150 s period that preceded each block. The Pearson Chi-square statistic was used to assess differences in the proportions of responsive neurons.

#### Ensemble Analyses: PCA

Principal Component Analysis (PCA) was performed on neurons in single sessions or on all 934 neurons from the block sessions using the *pca* command in Matlab. In order to align the sessions having different block orders and ITIs, neuron × time matrices (binned at 1 s) were constructed that were aligned to tone onset with the ITIs truncated to 30 s. Trials were concatenated to preserve presentation order and the resultant spike count vectors were then z-scored. Multiple single unit activity (MSUA) spaces were constructed using time bins from all neurons. As in our past studies (Lapish et al., 2008; Durstewitz et al., 2010; Hyman et al., 2012; Ma et al., 2016), the regularized Mahalanobis distance (D_Mah_) was calculated between clusters of points in the full, non-reduced MSUA space. The same number of units and time bins were used for each distance calculation by taking the smallest recorded ensemble size and randomly selecting this number of neurons in larger ensembles 100× and averaging the results. Control distances were calculated in the same manner but using blocks of equal size that contained random permutations of time bins extracted from throughout the session. This procedure was also performed 100x and the results averaged. Since the full dimensional MSUA spaces could not be visualized, the dimensionality was reduced from 1 to 3 dimensions by PCA or t-Distributed Stochastic Neighbor Embedding (t-SNE; van der Maaten and Hinton, 2008) using code implemented in Matlab (van der Maaten, 2010). PCA was performed prior to the application of the t-SNE algorithm which then used the top 5–20 PCs. Perplexity was tested between 10 and 1000× with best visual separation typically obtained at 50. For the mixed-trial sessions, t-SNE reduced the dimensionality to 3 and included only sessions with >30 neurons (5/8 sessions). Prior to performing t-SNE the ITIs were truncated at −30 s from the onset of the current trial and the spike counts for each neuron were averaged across each of the nine trials types depicted. For the statistical comparisons of distances, t-SNE (1 dimensional) or D_Mah_ was performed individually on the eight sessions and the distances for each of the nine trial types were calculated and the results compared by way of an ANOVA and *post hoc* tests. In order to test the effects of movement on ensemble block patterns, the animal’s xy tracking data was used to construct a movement vector (change in position between adjacent 200 ms time bins) that served as a factor in a linear regression model. The resultant residual matrix gave the portion of the spike count in each bin that was not accounted for by movement. The residual terms from all neurons were then combined to form a matrix. The raw and residual spike count matrices were truncated to the 30 s surrounding the tones and PCA was performed independently on each.

## Results

### Differences in Movement Across Blocks

Experiments involved delivering either a food pellet, a brief shock or nothing after a unique tone was delivered. The tone that preceded delivery of a food pellet was termed the F-tone and the block where the F-tone and food was delivered was termed the F-block. Likewise for the tone and block associated with the shock (i.e., the S-tone and S-block) and the tone and block associated with the null outcome (i.e., the N-tone and N-block).

The behavioral analysis characterized movement differences during the tone and outcome periods of each block. Samples of 10 trial paths from a single session are shown in Figure 1A. Trajectories during the F-block (green) tended to traverse in the direction of the food cup, whereas trajectories in the S-block (red) were smaller in amplitude. Two different statistical tests were performed on the displacement data obtained from movement tracking videos. The first test was designed to identify significant changes in movement displacement that occurred at the time of the tone or outcome within each block (Figure 1B). A series of Kolmogorov-Smirnov (KS) tests were performed that compared the 5 s baseline period with 1 s intervals throughout the tone and outcome periods. Movement during the N-tone tone did not differ from the baseline period. A significant increase in movement was observed 2–3 s into the F-tone while an increase in movement occurred for the first 1 s of the S-tone. Significant increases in movement relative to the baseline periods also occurred during the outcome epochs of the F-block and S-block (Figure 1B). The second test sought to determine whether differences in movement extended past the tone/outcome periods. An ANOVA compared the mean number of pixels traversed during the inter-trial periods of each block. This revealed a significant overall effect of block (*F*_(2,27)_ = 8.04 *p* = 0.0018); N-block, $\bar{x}$ = 1.45 ± 0.9 pixels/s; F-block $\bar{x}$ = 1.89 ± 0.57 pixels/s; S-block $\bar{x}$ = 0.73 ± 0.47 pixels/s; Figure 1B) while *post hoc* analyses indicated that significantly more pixels were traversed during the ITI of the F-block than the S-block (*p* = 0.0014) whereas the N- and F-blocks did not differ from each other (*p* = 0.3665). There was the most movement in the S-block at tone and shock onset but the least amount of movement during the inter-trial periods, owing to a startle response that was followed by freezing. The F-block had the most movement overall with an increase at tone onset and another at food delivery as the animal oriented and moved to obtain the food. These analyses indicated that the animals were responding in a manner consistent with the emotional reaction we hoped to evoke in each block.

### Block Specific Tone and Outcome Responses of Single Neurons

In total 934 neurons were recorded from four animals across 15 block sessions. The approximate locations of the tetrodes are shown in Figure 2 while examples of how neurons responded to the tones and outcomes are shown in Figure 3. Individual neurons could respond to either one, two or three of the tones or outcomes in an excitatory or inhibitory manner (Figure 3). Some neurons exhibited transient responses at the time of the tones or outcomes across the three blocks (top rows), some primarily exhibited marked differences in background firing (bottom rows) while others showed mixtures of these two profiles (middle rows).

In order to quantify the tone responses, the spike counts of each neuron during the initial 2 s of the tone period were tested against spike counts during the 10 s period preceding tone onset, using a KS test. In total, 362/934 neurons were identified as “tone responsive” using this measure (Figure 4). A larger proportion of neurons were found to be responsive to the S-tone (*n* = 237/362) than the F-tone (*n* = 102/362, *χ*^2^ = 124.69, *p* < 0.0001) or the N-tone (*n* = 131/362; *χ*^2^ = 99.76, *p* < 0.0001). Overall, 25% (*n* = 91) of tone responsive neurons were responsive to more than one tone. Firing during the initial 3 s of the outcome period was also compared to firing during the same 10 s pre-tone period and in this case, 410/934 neurons were identified as “outcome responsive” (Figure 4) of which 28% (*n* = 115/410) were found to be responsive to more than one outcome. As with the tones, a larger proportion of neurons were responsive to the foot shock outcome (*n* = 270/410) as compared to the food outcome (*n* = 153/410, *χ*^2^ = 154.97, *p* < 0.0001) or the null outcome (*n* = 122/410, *χ*^2^ = 270.80, *p* < 0.0001). Overall, 36.7% of neurons that were tone responsive were also outcome responsive, however single neurons were not necessarily responsive to the same tone and outcome.

### Block Specific Background Responses

When zooming out from the tone/outcome periods, clear block differences become apparent (Figure 5). The activity of some neurons changed gradually, while others changed abruptly across blocks. PCA was used to visualize the main patterns of ensemble activity that emerged across all 934 neurons (Figure 6). PC1 and PC2 which together accounted for 46.5% of the total variance, captured the large block offsets that dominated the rasters in Figure 5. Note that PC1 largely separated the blocks based on valence, as it took on high positive values for the S-block and negative values for the F-block, with the N-block in between. PC3 and PC4 exhibited transient responses around the tone and outcome periods, especially for the S-block. The ranked loadings of all the neurons on the top four PCs and the variance accounted for, are also given in Figure 6A. PCA therefore indicated that the steady-state, block-dependent shifts in ensemble dynamics dominated over the more transient changes evoked by the tones/outcomes.

PCA blindly decomposed ensemble activity in a manner that was unconstrained by the task structure. A second approach was therefore used to quantify how the ensembles segregated the different task epochs. As in our past studies, the spike counts of the neurons were plotted as separate axis in a MSUA space (Lapish et al., 2008; Durstewitz et al., 2010; Ma et al., 2016) where each point in the space represented the normalized spike counts of all neurons during a single time bin. Figure 6B plots an MSUA space from a single session with the color of each point denoting the block and the shape of the point denoting the task epoch. In general, points of the same color tended to cluster together in the space. This is notable since the points in each cluster were derived from time bins associated with very different events, including tones (diamonds), outcomes (squares) and ITIs (dots). The regularized Mahalanobis distance (D_Mah_) between the clusters of points in the full dimensional, non-reduced MSUA space were calculated for each session and compared by means of an ANOVA. The average D_Mah_ between tone clusters associated with different blocks was larger than the D_Mah_ between tone and outcome clusters derived from a single block (*F*_(6,78)_ = 7, *p* < 0.0001; Figure 6C). *Post hoc* tests revealed that all block to block distances were larger than all within-block comparisons (*p* < 0.0001; Figure 6C). These analyses confirmed what is obvious from the panels above, namely that the ensembles parsed the task primarily by block. It also illustrates how strongly the block representations affected the way that the tones and outcomes were encoded.

### Transitions Between Valence-Specific Patterns

It is difficult to tell from the above analyses exactly when the shift to a given activity pattern occurred. To address this question, the full MSUA space was reduced to a single dimension using t-SNE (van der Maaten and Hinton, 2008). Because t-SNE maintains the t-distributed distances between the points in the high dimensional space, differences in 1D t-SNE values provide a measure of the relative changes in ensemble activity patterns across time. This allowed us to visualize how the distances between points (i.e., the differences in ensemble activity) evolved. In the individual session shown in Figure 7A, the shifts tended to be quite abrupt but occurred at different times for each block. The shift occurred during the very first tone of the S-block and N-block, but was delayed by a few trials in the F-block. An abrupt shift was consistently locked to the first tone of the S-block in every session and was therefore clearly evident when the neurons from all sessions were combined (Figure 7B). On the other hand, the variability in the timing of the shift for the F- and N-blocks in different sessions produced the appearance of a gradual change in the combined ensemble (Figure 7B). Thus, the aversive cue produced a much more abrupt, consistent and earlier change in ACC ensemble dynamics than the appetitive or neutral cues and outcomes on this task.

These shifts could potentially be due to factors other than valence encoding however. For instance, if a tetrode were to shift during the session, the characteristics of the spike waveform recorded on one or more wires would change. If these changes were large enough, it would give the impression that one neuron shut off and a new neuron suddenly started to fire. If this occurred at block transitions, it could shift the ensemble dynamics in the MSUA space in a manner similar to that shown above. For this reason, we carefully monitored the waveform characteristics to ensure they remained stable throughout the session. Note in the example given in Figures 8A,B, that although there was some spread in the peak amplitudes, these amplitudes remained stable throughout the session. Also note that that the density of the points changed at various points, indicating that the neurons fired more in some periods of the session than others. It was this behavior that gave rise to the shifts described above.

A second issue to consider is that since we already determined that the animal’s movement patterns differed by block (Figure 1), it raised the possibility that the block-specific ensemble patterns might simply have been due to neurons tracking these differences in movement. This issue could not be addressed using the combined ensemble since the individual movements obviously differed by session. Instead the location tracking data was used as a single factor in a linear regression model for each neuron in each session. This yielded a residual term which represented the portion of a neuron’s firing that was not linearly related to this movement factor. PCA was then performed separately on the raw and residual spike count matrices. The profiles of the resultant PCs were very similar but not identical in the two cases (Figures 8C,D), indicating that while movement differences contributed, they could not fully account for the block-dependent differences in ensemble patterns.

**Figure 9:**
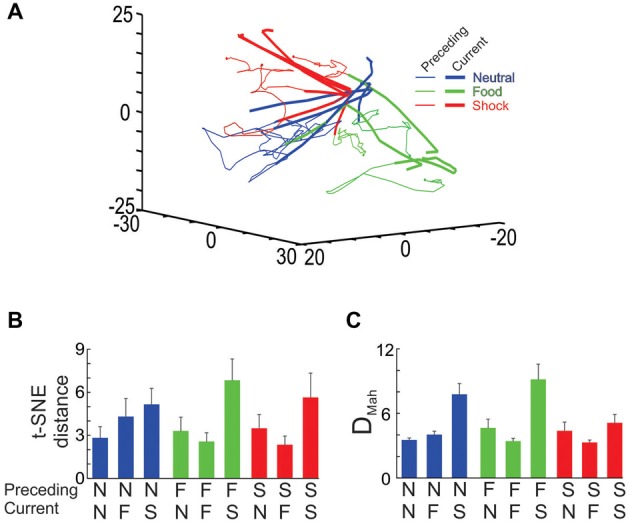
Ensemble transitions during the interleaved trial sessions. **(A)** The full dimensional MSUA space was reduced to 3 dimensions using t-SNE. Nine types of trials were identified and plotted separately. In each case, the time bin associated with the end of the outcome period of the preceding trial was denoted by a dot and the distance in MSUA space from this point, through the ITI and into tone period on the subsequent trial was plotted as a vector in the space. The tone/outcome period of the current trial was denoted by the thickening of the line vector. The dots and thin lines were colored based on the type of outcome on the preceding trial (food outcome = green, shock outcome = red, null outcome = blue), whereas the thickened line segments were colored based on the type of tone/outcome on the current trial (F-block = green, the N-block = blue, S-block = red). **(B)** The MSUA space was reduced to one dimension using t-SNE and the distance traversed by each of the nine vectors shown in **(A)** were calculated. The bars were colored as in **(A)** based on the preceding trial (N = neutral trial, F = food trial, S = shock trial). **(C)** The D_Mah_ between the last 5 s of the preceding trial and the 5 s tone period of the current trial were calculated for the same nine trial types and plotted in the same way. In both **(B,C)** the calculations were performed on each session separately and the averages (and SEM) are plotted.

Due to the nature of the blocked trials, each transition to a valence-specific pattern occurred only once per session. To investigate the shifts in greater detail, an interleaved trial design was employed where the three tones/outcomes were delivered in a randomized fashion. Figure 9A plots the average activity-state vectors starting from 15 s after the outcome of one trial, through the ITI and into the tone and outcome period of the subsequent trial. The thin lines denote the ITI period and were colored according to the valence of the preceding trial. The thick lines denote the tone and outcome periods of the current trial and were colored according to the valence of the current trial. Given that each vector spanned two trials and that each trial could involve one of three outcomes, there were nine trial-to-trial conditions. Note that regardless of the exact trial-to-trial condition, all line segments of a given color tended to cluster in the space. This indicated that each of the tones/outcomes evoked a consistent, valence-specific pattern regardless of when in the session it occurred. Also note that the thin lines were largely circumscribed within a given cluster, meaning that the valence-specific activity pattern from the preceding trial was largely maintained throughout the ITI. Finally note that if the valence of the current trial differed from the valence of the preceding trial, there was an abrupt redirection near the point where the lines thickened. These redirections moved the ensemble from the pattern associated with the preceding trial to the pattern associated with the current trial.

In order to test for trial order effects, we analyzed how the dynamics of the shifts differed across the nine trial-to-trial conditions. In the first analysis, the MSUA space was reduced to 1D using t-SNE and the distances traversed during each of the nine trial-to-trial conditions were calculated and compared by means of an ANOVA. This revealed a significant overall difference across the nine conditions (*F*_(8,71)_ = 2.47, *p* = 0.02; Figure 9B) as the ensembles were found to transition through a greater distance in MSUA space whenever the S-tone was presented (i.e., N-S and F-S trials) as compared to when the N-tone (i.e., N-N and F-N trials; *p* < 0.005) or the F-tone (N-F and F-F trials, *p* < 0.005) was presented. The results were similar when we repeated the analysis by calculating the D_Mah_ in the full dimensional MSUA space between the clusters associated with the last 5 s of the ITI of one trial and the subsequent tone period of the next trial (Figure 9C). Both analyses led to the same conclusion, that the ensembles underwent a larger shift whenever an aversive trial was presented, regardless of the preceding trial type.

## Discussion

The present study investigated the responses of ACC neurons and ensembles to tones conditioned to outcomes of positive, neutral or negative affective valence. Behavioral analyses revealed differences in movement that were consistent with the affective characteristics of the three blocks. The S-block had the largest impact on neuronal activity, but single neurons exhibited responses to mixtures of tones and/or outcomes that were not necessarily restricted to a single valence. Block-specific ensemble patterns emerged early in each block and remained in place throughout the remainder of the block. During the interleaved sessions, the ensembles shifted from representing the prior outcome to the current outcome at the start of each new trial and the shift was larger if the current outcome was aversive.

The block specific tone and outcome responses of individual neurons (Figure 2) are consistent with an extensive literature describing how MFC/ACC neurons encode aversive events and associated behaviors (Powell et al., 1990, 1996; Sikes and Vogt, 1992; Baeg et al., 2001; Wang et al., 2004; Gilmartin and McEchron, 2005; Laviolette et al., 2005; Powell and Ginsberg, 2005; Sikes et al., 2008; Burgos-Robles et al., 2009; Zhang et al., 2011; Ning et al., 2013; Halladay and Blair, 2015; Dejean et al., 2016) as well as the separate literature showing that MFC/ACC neurons respond robustly to rewards and reward-associated cues and actions (Shidara and Richmond, 2002; Kennerley et al., 2009; Hayden and Platt, 2010; Rushworth et al., 2011; Wallis and Kennerley, 2011). Yet very few animal studies have directly compared responses of ACC neurons to aversive and appetitive events. To our knowledge, Koyama et al. (2001) was the first to do so and of the 77 valence-discriminatory neurons recorded in that primate study, 34 showed significant activity during a shock-associated cue while 13 responded to a food cue, with 30 neurons being responsive to both. A similar pattern was observed in the present study in that the largest proportion of neurons was responsive to the S-tone and outcome but of the neurons responsive to the F-tone, 38% were also responsive to the S-tone and of the neurons responsive to the food, 42% were also responsive to the shock.

The most striking finding was the large block offsets that were evident in the PCs and the rasters of many individual neurons that dwarfed the changes evoked by the cues and outcomes. Tonic responses to aversive stimuli of a shorter duration have been reported in past studies. Laviolette et al. (2005) observed that MFC neurons exhibited increases in spontaneous activity and burst firing for many seconds after conditioned stimulus (CS) offset. Burgos-Robles et al. (2009) reported that ~26% of MFC neurons showed increased activity throughout and for up to 10 s after a 30 s CS that preceded a foot-shock while Halladay and Blair (2015) reported that if the expected aversive outcome did not follow a CS, some of the MFC neurons activated by the CS remained activated for over a minute. Such tonic changes are not limited to aversive conditioning as Takehara-Nishiuchi and McNaughton (2008) found that 40% of MFC neurons changed their baseline firing rate on trials where a CS was paired with food relative to trials where the CS was presented alone. ACC neurons can also maintain the representation of past outcomes (reward or non-reward) until the start of the next trial or even across a few trials (Seo and Lee, 2007; Narayanan and Laubach, 2008; Bernacchia et al., 2011; Hyman et al., 2017). Finally, in Monosov and Hikosaka (2012), neurons located nearby in the ventromedial frontal cortex maintained differential firing throughout valenced blocks of up to 5 min duration.

Our results expand on these studies by demonstrating that valence-specific activity patterns can persist for tens of minutes if the valenced event is repeated at regular intervals. In fact it appeared that the ensemble pattern evoked by an outcome slowly decayed away until an event of a different valence occurred. In the block sessions a consistent pattern dominated the network until the start of a new block, which meant that the ensemble pattern evoked by the first tone (or first few tones and outcomes) remained in place for the next 15–30 min, including all the ~50 s ITI periods where no tones or outcomes were delivered. This did not however mean that all the neurons exhibited elevated firing throughout the entire block. In fact, we typically find that ~10%–20% of ACC neurons are active at any one time (Ma et al., 2016). The nature of the ensemble patterns is such that whenever a group of neurons become active, they tend to become active in the same way, even though any given neuron contributes to the pattern for only some fraction of the time.

The shift to the S-block pattern occurred during the first tone of the block, prior to the animal experiencing the first footshock of the session. This implies that through learning, the S-tone came to evoke an aversive reaction in the animal and we believe it was this reaction that was represented by the activity patterns we recorded. The same was true of the activity pattern associated with the F-block, but in this case the reaction was appetitive. A distinct activity pattern was also observed during the N-block which was ostensibly neutral but the animals were likely reacting to the absence of shock or food, making it motivationally relevant as well.

There were important differences in the responses recorded across blocks. More individual neurons were responsive to the S-tone and shock than to the other tones or outcomes (Figure 4). The shift to the F- and N-block patterns were also slower and more variable across sessions than was the case for the S-block (Figure 7). Furthermore, the shifts in ensemble dynamics during each trial of the interleaved sessions were always largest whenever the current trial was aversive (Figure 9). This could be taken as evidence that the ACC “prefers” aversive events over neutral or appetitive events. However, it is also possible that the aversive trials evoked the largest responses because these trials were the most arousing. Although arousal is a difficult factor to control for, prior to the recordings we tested different shock intensities to find the minimal required to evoke a behavioral reaction in each animal. Furthermore, rats were restricted to 90% of their free-feeding weight and were not fed in the 12 h preceding each session to ensure they were motivated by food rewards. For these reasons, we assumed that if anything, the food trials would engender the greatest arousal. If instead we assume that the aversive trials were the most arousing and the neurons were mainly tracking arousal, then it should have been possible to find a PC that strongly correlated with the presumed differences in arousal across blocks. Such a PC would exhibit large deviations during the S-block, smaller deviations during the F-block and assume near zero values during the N-block. In none of the top PCs did such a ranking appear. This does not mean that arousal was not an important factor, but only that it did not appear to be the strongest factor. The most likely explanation for the present results was that the responses were driven by some combination of movement, valence and arousal. Russell and Barrett (1999) and Duncan and Barrett (2007) proposed that the basis of all emotion is core affect which has two dimensions, hedonic valence (pleasant/unpleasant) and arousal (high/low). By integrating information about core affect states with information about events and actions, the ACC may signal how strongly to react to events and experiences.

## Author Contributions

JS conceived of the project, analyzed the data and wrote the article. BC conducted the experiments and helped with data analysis. JG helped with surgeries and spike sorting.

## Conflict of Interest Statement

The authors declare that the research was conducted in the absence of any commercial or financial relationships that could be construed as a potential conflict of interest.
